# Transcription factors early growth response gene (Egr) 2 and 3 control inflammatory responses of tolerant T cells

**DOI:** 10.1002/iid3.210

**Published:** 2018-01-03

**Authors:** Becky Omodho, Tizong Miao, Alistair L.J. Symonds, Randeep Singh, Suling Li, Ping Wang

**Affiliations:** ^1^ The Blizard Institute, Barts and The London School of Medicine and Dentistry Queen Mary University of London 4 Newark Street, London UK; ^2^ Bioscience Brunel University London Kingston Lane, London UK

**Keywords:** Egr2, T cells, tolerance

## Abstract

**Introduction:**

Impaired proliferation and production of IL2 are the hallmarks of experimental T cell tolerance. However, in most autoimmune diseases, auto‐reactive T cells do not display hyper proliferation, but inflammatory phenotypes.

**Methods:**

We have now demonstrated that the transcription factors Egr2 and 3 are important for the control of inflammatory cytokine production by tolerant T cells, but not for tolerance induction.

**Results:**

In the absence of Egr2 and 3, T cell tolerance, as measured by impaired proliferation and production of IL2, can still be induced, but tolerant T cells produced high levels of inflammatory cytokines. Egr2 and 3 regulate expression of differentiation repressors and directly inhibit T‐bet function in T cells. Indeed, decreased expression of differentiation repressors, such as Id3 and Tcf1, and increased expression of inflammatory transcription factors, such as RORγt and Bhlhe40 were found in Egr2/3 deficient T cells under tolerogenic conditions. In addition, T‐bet was co‐expressed with Egr2 in tolerant T cells and Egr2/3 defects leads to production of high levels of IFNγ in tolerant T cells.

**Conclusions:**

Our findings demonstrated that despite impaired proliferation and IL2 production, tolerant T cells can display inflammatory responses in response to antigen stimulation and this is controlled at least partly by Egr2 and 3.

## Introduction

Tolerance to self‐antigens is an important function of peripheral T cells. In homeostatic conditions, self‐tolerance of T cells is controlled by extrinsic mechanisms such as regulatory T (Treg) cells 1 and also intrinsic mechanisms induced by suboptimal stimulation of T cell receptors (TCR) in response to self‐antigens 2. In optimal immune responses against pathogen infection, proliferation and production of IL2 are essential for clonal expansion of antigen specific T cells, which is coupled with differentiation and acquisition of effector function 3. Under tolerogenic conditions, the TCR signaling pathway is only partially activated rendering T cells unable to express IL2 and cell cycle regulatory molecules 2. Therefore, the hallmarks of tolerant T cells are impaired proliferation and production of IL2 2. However, although hypoproliferation and reduced production of IL2 in response to antigen stimulation are the hallmarks of T cell tolerance, auto‐reactive T cells from autoimmune disease patients rarely display hyperproliferation in response to autoantigens. On the contrary, T cells from autoimmune disease patients often show dysfunctional responses to TCR stimulation with defects in proliferation and production of IL2 4, 5. Despite impaired proliferation, the autoreactive T cells from these patients are generally hyperactivated and inflammatory 4, 5, indicating that self‐tolerance is not limited to the control of proliferation and production of IL2, but also requires control of the inflammatory differentiation of effector T cells 6.

Egr2 and 3 are transcription factors induced in tolerant T cells in response to TCR stimulation 7, 8, 9. T cell‐specific deficiency of Egr2 and 3 leads to loss of self‐tolerance and the development of severe autoimmune symptoms 10, 11, 12. Interestingly, although CD2‐specific Egr2 and 3 deficient mice developed severe systemic autoimmune syndromes, Egr2 and 3 deficiency does not enhance TCR mediated proliferation. In contrast, Egr2 and 3 deficient T cells proliferated poorly and were defective in IL2 production in response to TCR stimulation 11. Thus, Egr2 and 3 are not inhibitors of TCR mediated proliferation, but promote T cell proliferation. Importantly, these findings demonstrated that autoimmune responses do not necessarily require hyper‐proliferation and excessive production of IL2 by autoreactive T cells, but can result from hyper‐activation and inflammatory responses, which resemble the dysfunctional T cells in autoimmune diseases such as lupus 4, 5.

To investigate the function of Egr2 and 3 in tolerant T cells, we assessed the role of Egr2 and 3 in the development of T cell tolerance in vitro and in vivo. Tolerance, as measured by impaired proliferation and IL2 production, can be induced in Egr2 and 3 deficient T cells. However, despite proliferative tolerance, Egr2 and 3 deficient tolerant T cells expressed high levels of CD44 and CD69, and produced high levels of inflammatory cytokines in response to antigen stimulation. We found that together with Egr2, T‐bet was also induced in tolerant T cells indicating a repressive effect of Egr2 and 3 on T‐bet function in tolerant as well as effector T cells 13. We also found that Egr2 and 3 controlled regulators of differentiation 14 are defective in tolerant T cells from CD2‐specific Egr2 and 3 deficient mice. Our findings not only defined the role of Egr2 and 3 in tolerant T cells, but also revealed a novel tolerance mechanism that inhibits the effector function of tolerant T cells.

## Experimental Procedures

### Mice

CD2‐Egr2/3^−/−^ (CD2‐Cre/Egr2^loxp/loxp^/Egr3^−/−^) and GFP‐Egr2 knockin mice were reported previously 11, 14. C57BL/6 mice purchased from Charles River were used as controls in all experiments. All mice analyzed were 7–8 weeks of age. No animal was excluded from the analysis, and the number of mice used was consistent with previous experiments using similar experimental designs. All mice were maintained in the Biological Services Unit, Brunel University, and used according to established institutional guidelines under the authority of a UK Home Office project license.

### Antibodies and flow cytometry

Fluorescein isothiocyanate (FITC)‐conjugated antibodies to CD4 (cat 11‐0041‐81, clone GK1.5), CD45.1 (cat 11‐0453‐81, clone A20), and IFNγ (cat 11‐7311‐81, clone XMG1.2); phycoerythrin (PE)‐conjugated antibodies to CD3 (cat 12‐0031‐81, clone 145‐2C11), CD4 (cat 12‐0041‐81, clone GK1.5), CD25 (cat 12‐0251‐81, clone PC61.5), CD62L (cat 12‐0621‐81, clone MEL‐14), CD69 (cat 12‐0691‐81, clone H1.2F3), and Egr2 (cat 12‐6691‐80, clone erongr2); PerCP‐Cy5.5 labeled antibodies to CD3 (cat 45‐0031‐80, clone 145‐2C11) and CD45.2 (cat 45‐0454‐80, clone 104); allophycocyanin (APC)‐conjugated antibodies to CD44 (cat 17‐0441‐81, clone IM7), CD3 (cat 17‐0031‐81, clone 145‐2C11), Egr2 (cat 17‐6691‐82, clone erongr2); and PEcy7‐conjugated antibodies to CD44 (cat 25‐0441‐81, clone IM7) and T‐bet (cat 25‐5825‐82, clone 4B10) were obtained from E‐bioscience. FITC or PE labeled antibodies to TCRVβ3 (clone KJ25, cats: 553208 and 553209) were purchased from BD Biosciences. Antibodies to CD3 (cat 557306, clone 145‐2C11) and CD28 (cat 557393, clone 37.51) for stimulation and 7AAD were obtained from BD Biosciences. For flow cytometry analysis, single cell suspensions were analyzed on a LSRII or Canto (BD Immunocytometry Systems) and the data were analyzed using FlowJo (Tree Star). Cell sorting was performed on a FACSAria sorter with DIVA option (BD Immunocytometry Systems).

### Cell isolation and stimulation

Naïve CD4^+^ T cells were purified by negative selection using a MACS system (Miltenyi Biotec) or isolated by sorting CD25^−^CD4^+^CD44^high^CD62L^−^ and CD25^−^CD4^+^CD44^low^CD62L^+^ T cells by FACS. Cells were gated on CD25^‐^ cells to exclude activated cells and Treg. Purified CD4^+^ T cells were stimulated with plate‐bound anti‐CD3 at 1 µg/mL, or at the indicated concentrations, together with anti‐CD28 (2 µg/mL) antibodies. For analysis of Egr2 and T‐bet expression, the cells were processed using the Foxp3 staining kit (E‐bioscience). For analysis of cytokine producing cells, the cells were stimulated with 50 ng/mL PMA and 200 ng/mL Ionomycin in the presence of Golgistop (BD) for 4 h before analysis of cytokine producing cells by flow cytometry.

### Anergy induction

Naïve CD4 T cells were stimulated with 1 µg/mL anti‐CD3 for 24 h. The cells were then washed with PBS and rested for 24 h in fresh medium. After resting, cells were recounted and re‐stimulated using plates coated with anti‐CD3 (at 1 µg/mL or the indicated concentrations) and anti‐CD28 (2 µg/mL).

### Proliferation

For in vitro anergy, 2 × 10^5^ cells/well were re‐stimulated with anti‐CD3 at the indicated concentrations and anti‐CD28 (2 µg/mL) in triplicate for 3 days. For SEA responding T cells, isolated CD4^+^TCRVβ3^+^ cells were incubated with SEA loaded congenic DC cells at a 1:1 ratio in 96‐well plates in triplicate for 3 days. A total of 1 μCi of [3H]TdR was added for the last 8 h of culture, the cells were then harvested and subjected to scintillation counting to measure [3H]TdR incorporation.

### ELISA

The concentrations of IL2 and IFNγ in the cell culture supernatants were measured using ELISA kits (R & D) according to the manufacturer's instructions.

### SEA activation and tolerance

Ten micrograms of Staphylococcal enterotoxin A (SEA, Sigma–Aldrich) in 0.2 mL of phosphate‐buffered saline with 1% normal congenic serum were injected intravenously (i.v.) at 4 day intervals. SEA was injected five times to induce tolerance and once to induce T cell activation 15. Twenty‐four hours after the last injection, CD4^+^TCRVβ3^+^ cells were isolated by FACS and stimulated with SEA loaded dentritic cells (DC) at a 1:1 ratio in vitro. The SEA loaded DCs were prepared as follows: splenic CD11c^+^ cells from wild‐type mice were isolated by MACS and cultured in the presence of 10 ng/mL GM‐CSF (R & D) for 48 h to induce DC cell maturation. Mitomycin C (Sigma–Aldrich) at 50 µg/mL was added for the last 30 min of culture. DCs were washed and loaded with SEA by incubation with SEA at 200 ng/mL for 30 min. Unbound SEA was removed by washing and loaded DCs were used to stimulate CD4^+^TCRVβ3^+^ cells at a 1:1 ratio.

### Quantitative real‐time PCR

Total RNA was extracted from cells using Trizol (Invitrogen) and reverse transcribed using random primers (Invitrogen). Quantitative real‐time PCR was performed on a Rotor‐Gene system (Corbett Robotics) using SYBR green PCR master mix (Qiagen). The primers used are as follows: Egr2: sense 5′‐CTTCAGCCGAAGTGACCACC‐3′ and antisense 5′‐GCTCTTCCGTTCCTTCTGCC‐3′; Egr3: sense 5′‐GCTCTTCCGTTCCTTCTGCC‐3′ and antisense 5′‐CGGTGTGAAAGGGTGGAAAT‐3′; IL2: sense 5′‐GCATGTTCTGGATTTGACTC‐3′ and antisense 5′‐CAGTTGCTGACTCATCATCG‐3′; IFNγ: sense 5′‐CCATCAGCAACAACATAAGC‐3′ and antisense 5′‐AGCTCATTGAATGCTTGGCG‐3′; Id3: sense 5′‐ACATGAACCACTGCTACTCGC‐3′ and antisense 5′‐TGAGCTCAGCTGTCTGGATCG‐3′; Tcf1: sense 5′‐CCCAGCTTTCTCCACTCTACG‐3′ and antisense 5′‐CTGTGAACTCCTTGCTTCTGGC‐3′; Bhlhe40: sense 5′‐ACGTTGAAGCACGTGAAAGC‐3′ and antisense 5′‐GAAGTACCTCACGGGCACAA‐3′; RORγt sense 5′‐TGAGGCCATTCAGTATGTGG‐3′ and antisense 5′‐CTTCCATTGCTCCTGCTTTC‐3′; IL17: sense 5′‐AGCGTGTCCAAACACTGAGG‐3′ and antisense 5′‐CTATCAGGGTCTTCATTGCG‐3′; GAPDH: sense 5′‐TGCACCACCAACTGCTTAGC‐3′ and antisense 5′‐GGCATGGACTGTGGTCATGAG‐3′; IL6: sense 5′‐TGGTCTTCTGGAGTACCATAGC‐3′ and antisense 5′‐ACTCCTTCTGTGACTCCAGC‐3′; GM‐CSF: sense 5′‐TGGTCTACAGCCTCTCAGCA‐3′ and antisense 5′‐CCGTGACCCTGCTCGAATA‐3′.

The data were analyzed using the Rotor‐Gene Software. All samples were run in triplicate, and relative mRNA expression levels were obtained by normalizing against the level of Gapdh from the same sample under the same program using: relative expression = 2^(CTgapdh − CTtarget)^.

### Bone marrow chimeras

Bone marrow was collected from CD2‐Egr2/3^−/−^ (CD45.2^+^) or wild‐type C57BL/6 (CD45.1^+^) mice. For each chimera, 20 × 10^6^ cells of a 1:1 mixture of CD2‐Egr2/3^−/−^ and C57BL/6 bone marrow cells were transferred intravenously into lethally irradiated (two doses of 550 rads) wild‐type C57BL/6 (CD45.1^+^) recipients. Recipient mice were allowed 6–8 weeks for reconstitution.

### Statistics

To analyze the statistical significance of differences between groups, Kruskal–Wallis tests followed by pairwise comparisons using Conover tests, as implemented in the R package PMCMR 16, with Benjamini–Hochberg correction for multiple comparisons were used. Differences with a *p*‐value < 0.05 were considered significant.

## Results

### Egr2 and 3 are not required to induce tolerance in T cells

**Figure 1:**
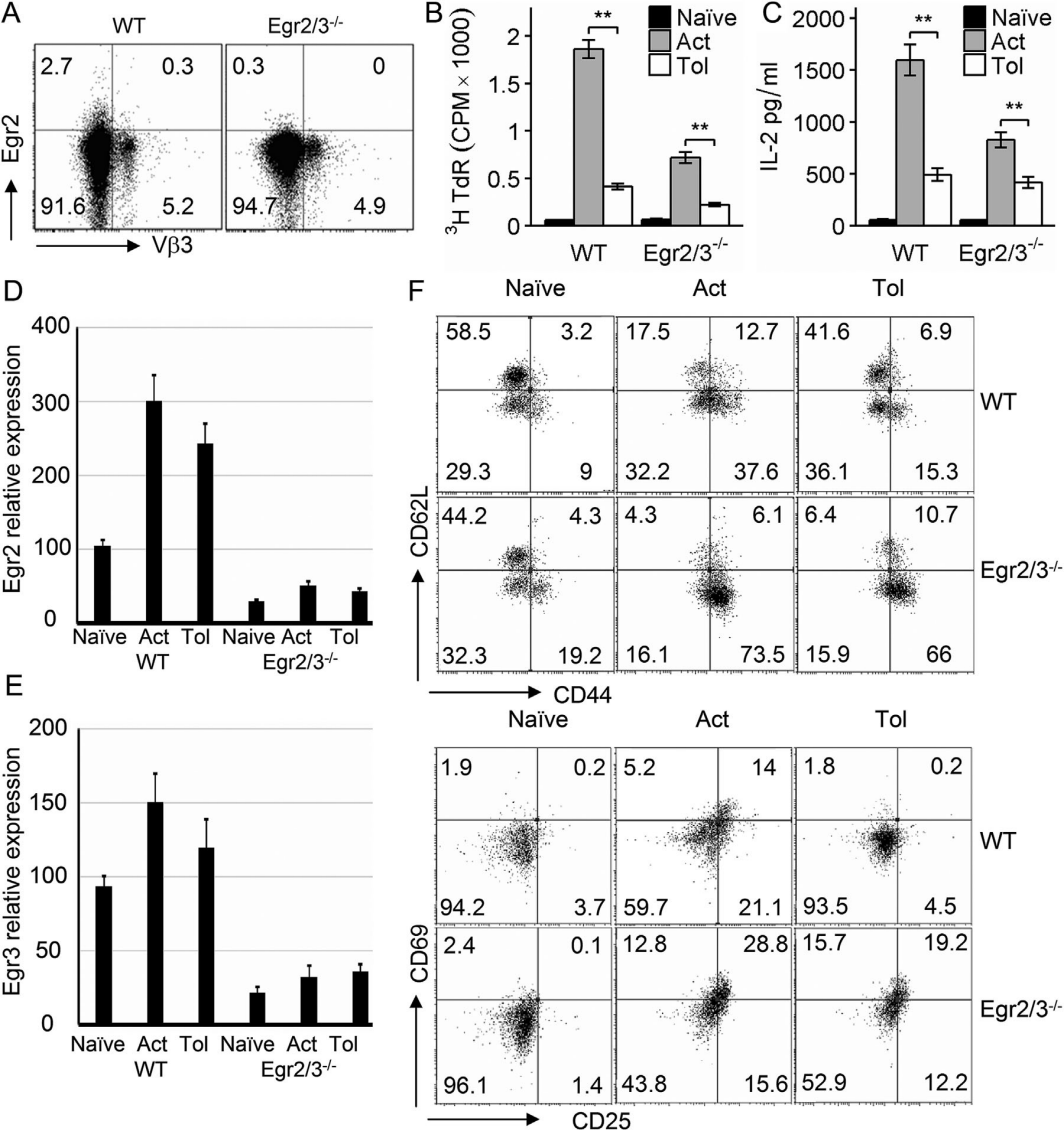
Egr2 and 3 are important for the control of the activation of tolerant T cells, but are not required for the induction of proliferative tolerance in vivo. (A) Egr2 and TCRVβ3 were analyzed on gated CD4 T cells from naive wild‐type (WT) and CD2‐Egr2/3^−/−^ (Egr2/3^−/−^) mice. (B–F) Wild‐type and CD2‐Egr2/3^−/−^ mice were injected with SEA once to activate T cells (Act) or five times with 4 day intervals to induce tolerance (Tol) and analyzed 24 h after the last injection. (B and C) TCRVβ3^+^CD4^+^ T cells were isolated and re‐stimulated in vitro with SEA loaded dendritic cells. Three days after re‐stimulation, proliferation (B) and IL2 in supernatants (C) were measured. (D and E) Expression of Egr2 and 3 in isolated TCRVβ3^+^CD4^+^ T cells was measured by RT‐PCR using the ddCt method with GAPDH as a reference gene. Data shown are the mean ± s.d. (F) Phenotyping of gated CD3^+^CD4^+^TCRVβ3^+^ cells. Data in A, D, E, F are from cells isolated from spleens and lymph nodes pooled from four mice in each group and are representative of three independent experiments. Data in B and C are from five mice in each group and are representative of three independent experiments. Data in B and C are the mean ± s.e.m. and were analyzed with Kruskal–Wallis tests followed by Conover tests with Benjamini–Hochberg correction. N.S. not significant, *p < 0.05, **p < 0.01.

Egr2 and 3 have been discovered in tolerant T cells induced in vivo by persistent antigen stimulation and in vitro by suboptimal T cell receptor stimulation 7, 8. It has been previously suggested that they play a role in the induction of T cell tolerance in vitro 8. However, in response to viral infection, Egr2 and 3 in T cells have been found to promote antigen induced proliferation 14. To assess whether Egr2 and 3 have a role in induction of T cell tolerance, TCRVβ3^+^CD4^+^ cells from wild‐type and CD2‐specific Egr2/3^−/−^ mice were analyzed after repeated administration of the super‐antigen Staphylococcal enterotoxin A (SEA); a well‐established model for induction of T cell tolerance in vivo 15. In naïve mice, the proportions of TCRVβ3^+^ cells among the CD4^+^ population were similar in wild‐type and CD2‐specific Egr2/3^−/−^ mice (Fig. 1A). SEA mediated activation of TCRVβ3^+^CD4^+^ T cells was analyzed in mice following a single injection of SEA. TCRVβ3^+^CD4^+^ T cells from wild‐type mice that received a single administration of SEA proliferated and produced IL2 in response to re‐challenge in vitro (Fig. 1B and C), while the proliferation and IL2 production of TCRVβ3^+^CD4^+^ T cells from SEA challenged CD2‐specific Egr2/3^−/−^ mice were reduced in response to SEA in vitro (Fig. 1B and C), consistent with previous findings 14. Repeated SEA stimulation induced tolerance of Vβ3^+^CD4^+^ T cells in wild‐type mice (Fig. 1B and C), consistent with previous findings 15, and Egr2 and 3 were induced in both activated and tolerant T cells in response to SEA stimulation (Fig. 1D and E). Similar to wild‐type mice, Vβ3^+^CD4^+^ T cells in CD2‐specific Egr2/3^−/−^ mice were also rendered tolerant by repeated administration of SEA (Fig. 1B and C). Crucially, despite the induction of tolerance, the tolerant Vβ3^+^CD4^+^ T cells from CD2‐specific Egr2/3^−/−^ mice were highly activated with high levels of CD25, CD44, and CD69 (Fig. 1F). These results demonstrate that Egr2 and 3 are not required for the TCR mediated induction of proliferative tolerance, but are important for controlling the activation of tolerant T cells.

**Figure 2:**
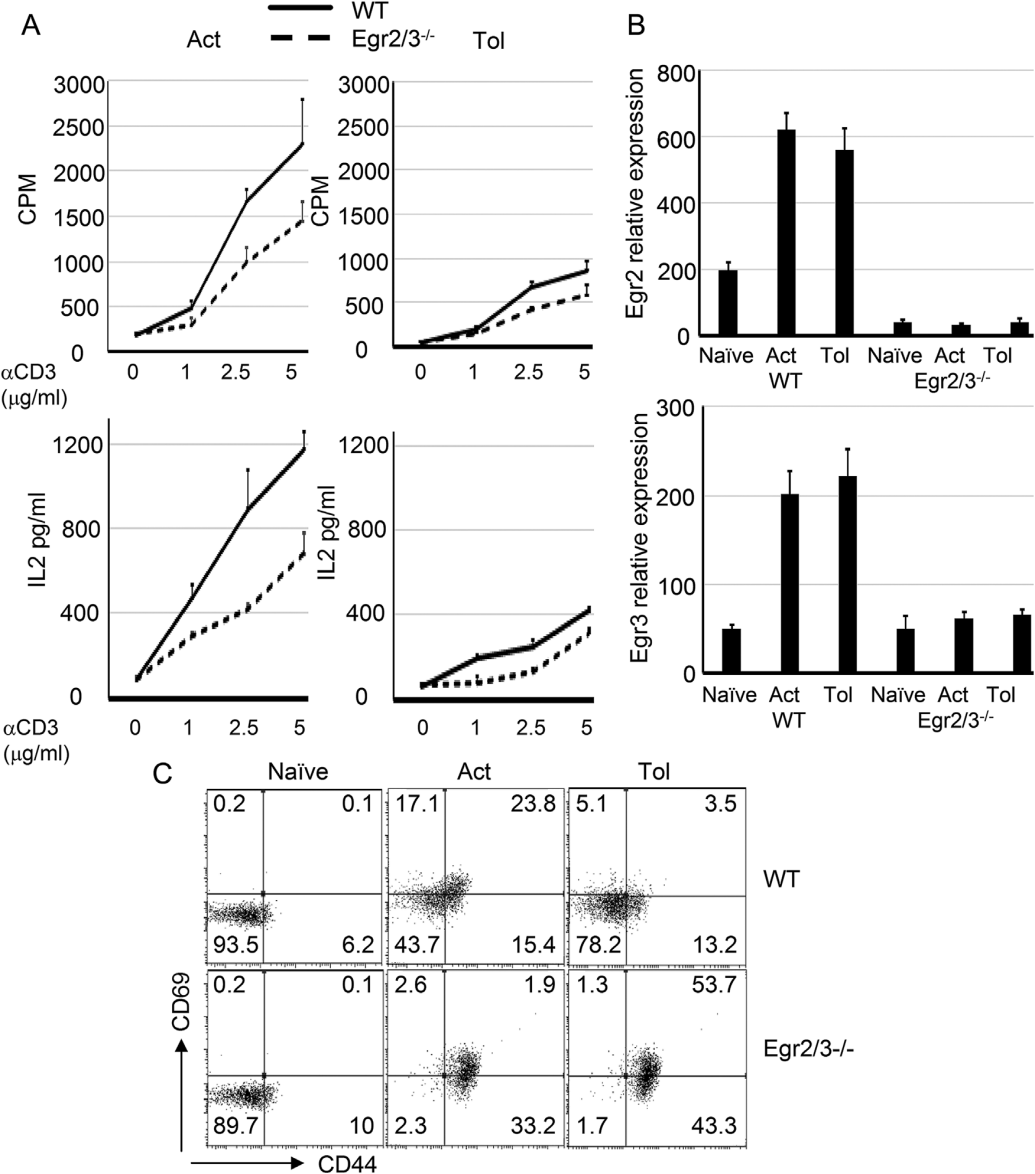
Egr2 and 3 are not required for the induction of T cell anergy, but control the activation of anergic T cells in vitro. (A) Naïve CD4 T cells (CD62L^+^CD44^lo^) from wild‐type (WT) and CD2‐Egr2/3^−/−^ (Egr2/3^−/−^) mice were stimulated with anti‐CD3 alone for 24 h, then washed and rested for 24 h before re‐stimulation with anti‐CD3 and anti‐CD28 (Tol). Proliferation (top panels) and IL2 production (bottom panels) were measured after 3 days and 24 h, respectively, and compared to activated T cells (Act). (B) Egr2 and 3 expression in naive, activated and tolerant T cells was measured by RT‐PCR using the ddCt method with GAPDH as a reference gene. Data shown are the mean ± s.d. (C) CD44 and CD69 expression on naïve CD4 T cells that were treated for 24 h with PBS (Naive), anti‐CD3 and anti‐CD28 (Act), or anti‐CD3 alone (Tol) was analyzed. Data are representative of three independent experiments.

The induction of T cell anergy in Egr2 and 3 deficient CD4 T cells was also analyzed in vitro by suboptimal T cell receptor stimulation in the absence of co‐stimulation. In addition to the impaired proliferative responses and IL2 production by Egr2 and 3 deficient T cells in response to optimal stimulation (Fig. 2A), consistent with our previous findings 11, anergy was effectively induced in Egr2 and 3 deficient CD4 T cells by stimulation with anti‐CD3 alone (Fig. 2A), indicating that although Egr2 and 3 are induced in both activated and tolerant T cells (Fig. 2B), they are not required for the induction of proliferative tolerance in vitro. In accordance with the over‐activation of Egr2 and 3 deficient tolerant T cells in vivo (Fig. 1F), Egr2/3 deficient anergic CD4 T cells were hyper‐activated as indicated by high levels of CD44 and CD69 (Fig 2C). Thus, Egr2 and 3 are not required for the development of T cell tolerance, but for controlling the activation of tolerant T cells.

### Egr2 and 3 are essential for the control of inflammatory responses of tolerant T cells

**Figure 3:**
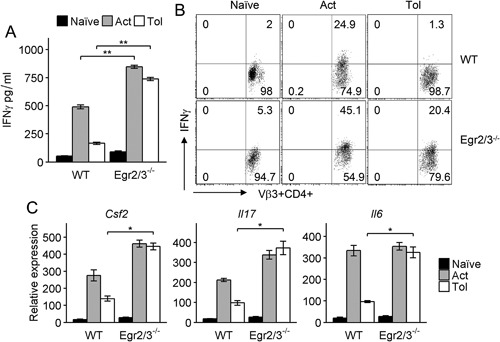
Deficiency of Egr2 and 3 results in production of inflammatory cytokines by tolerant T cells in vivo. Wild‐type (WT) and CD2‐Egr2/3^−/−^ (Egr2/3^−/−^) mice were injected with SEA once to activate T cells (Act) or five times with 4 day intervals to induce tolerance (Tol). Twenty‐four hours after the last injection, CD3^+^CD4^+^TCRVβ3^+^ cells were isolated and re‐stimulated in vitro with SEA loaded dendritic cells for 24 h. Supernatants were analyzed for IFNγ by ELISA (A) and IFNγ producing cells were quantified (B). Expression of GM‐CSF (encoded by *Csf2*), IL6 and IL17 in sorted CD3^+^CD4^+^TCRVβ3^+^ cells were analyzed by RT‐PCR using the ddCt method with GAPDH as a reference gene (C). Data are from five mice in each group and are representative of three experiments. Data in A and C are the mean ± s.e.m. and were analyzed with Kruskal–Wallis tests followed by Conover tests with Benjamini–Hochberg correction. N.S. not significant, *p < 0.05, **p < 0.01.

**Figure 4:**
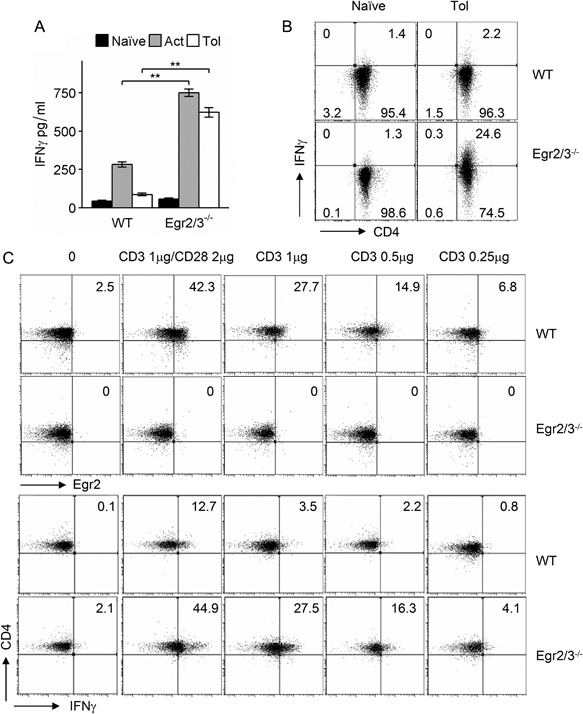
Egr2 and 3 are important to control inflammatory cytokine production by tolerant T cells. (A) Naïve CD4 T cells (CD62L^+^CD44^lo^) from wild‐type (WT) and CD2‐Egr2/3^−/−^ (Egr2/3^−/−^) mice were stimulated with anti‐CD3 alone for 24 h, then washed and rested for 24 h before re‐stimulation with anti‐CD3 and anti‐CD28 (Tol). IFNγ production was measured after 24 h and compared to naïve and activated T cells (Act). (B) After re‐stimulation, a proportion of tolerant T cells were treated with PMA and Ionomycin in the presence of Golgistop for 4 h and IFNγ producing cells were quantified. (C) Naïve CD4 T cells were isolated and stimulated with the indicated stimuli for 24 h and Egr2 (top) and IFNγ (bottom) expressing cells were analyzed. Data are representative of three independent experiments. Data in A are the mean ± s.e.m. and were analyzed with Kruskal–Wallis tests followed by Conover tests with Benjamini–Hochberg correction. N.S. not significant, *p < 0.05, **p < 0.01.

One of the major functions of Egr2 and 3 in T cells is to control the inflammatory responses of effector T cells 10, 11, 12, 13, 14. Although impaired proliferation and IL2 production are the hallmarks of tolerant T cells, the effector function of tolerant T cells has not been analyzed. We assessed the effector function of tolerant CD4 T cells from wild‐type and CD2‐specific Egr2/3^−/−^ mice by analysis of IFNγ production, a cytokine commonly produced by effector CD4 T cells. Compared to high levels of IFNγ production by Vβ3^+^CD4^+^ cells from SEA activated wild‐type and CD2‐specific Egr2/3^−/−^ mice, the IFNγ production in Vβ3^+^CD4^+^ cells from SEA tolerant wild‐type mice was significantly reduced (Fig. 3A and B). However, despite tolerance of proliferation and IL2 production, Vβ3^+^CD4^+^ cells from SEA tolerant CD2‐specific Egr2/3^−/−^ mice produced high levels of IFNγ (Fig. 3A and B). In addition to IFNγ, IL6, IL17, and GM‐CSF were also expressed in SEA tolerant Egr2/3 deficient CD4 T cells (Fig. 3C), indicating that Egr2 and 3 are not involved in induction of tolerance, but are important to control activation and effector function of tolerant T cells. A high level of IFNγ production was also detected in anergic CD4 T cells from CD2‐specific Egr2/3^−/−^ mice induced by suboptimal TCR stimulation (Fig. 4A and B).

To further investigate the association between the levels of Egr2 expression and IFNγ production, T cells were stimulated with different doses of anti‐CD3 with or without anti‐CD28 and Egr2 expression and IFNγ producing T cells were analyzed. Dose dependent Egr2 expression in wild‐type T cells corresponded well to the dose dependent proportion of IFNγ producing T cells among Egr2/3^−/−^ T cells (Fig. 4C), which further support the notion that Egr2 and 3 are important for controlling inflammatory responses in both optimal and suboptimal TCR stimulation. Taken together, these data indicate that although Egr2 and 3 are not required for the induction of proliferative tolerance, they are important for tolerance of effector function.

### Egr2 and 3 intrinsically control inflammatory, but not proliferative, responses of tolerant T cells

**Figure 5:**
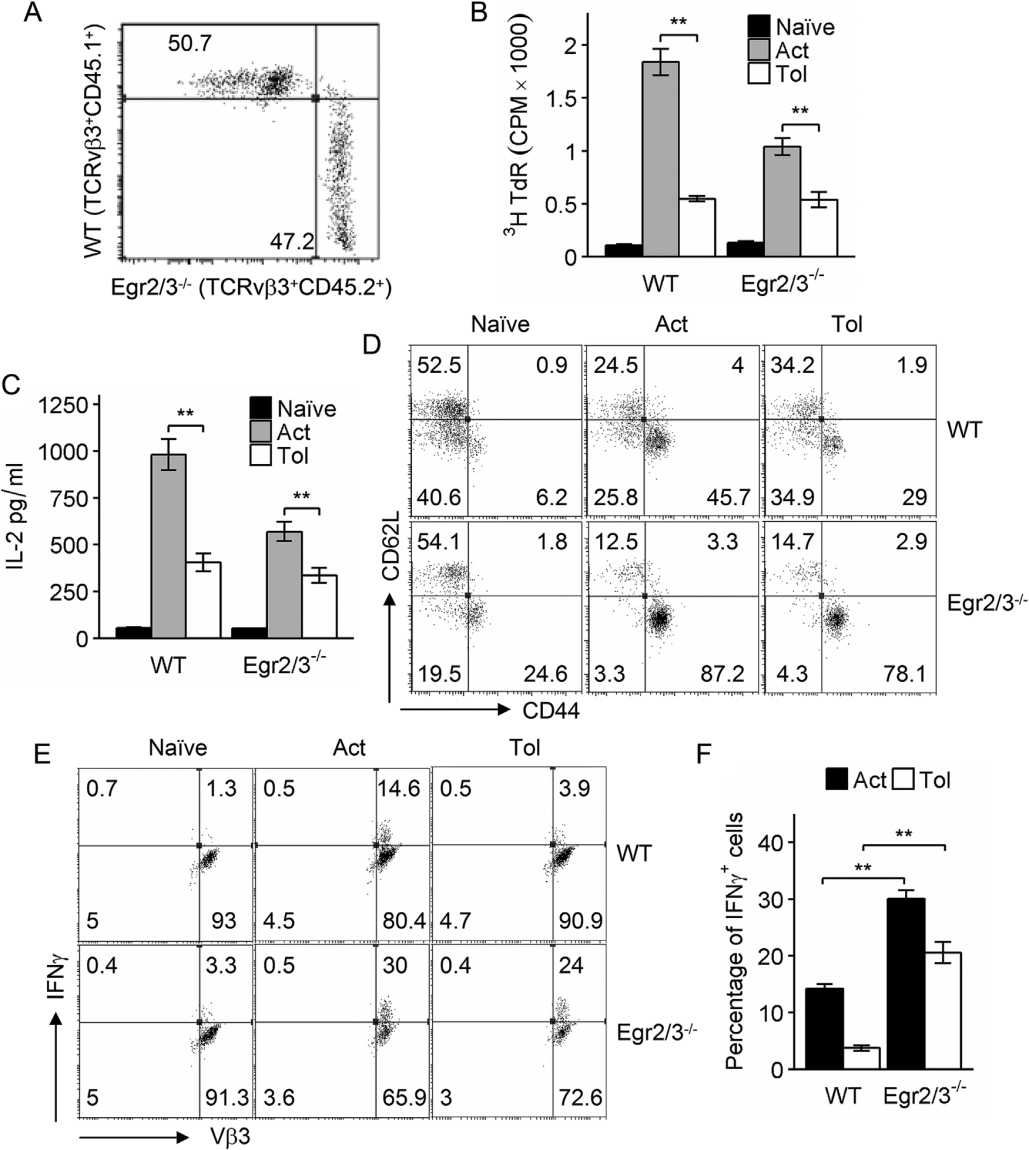
Egr2/3 intrinsically control inflammatory activation of tolerant T cells in vivo. An equal number of bone marrow cells from wild‐type (WT) and CD2‐Egr2/3^−/−^ (Egr2/3^−/−^) mice were adoptively transferred into irradiated wild‐type recipients. Six weeks after bone marrow reconstitution, wild‐type (CD45.1) and Egr2/3^−/−^ (CD45.2) cells among gated CD4^+^TCRVβ3^+^ cells were quantified (A). Recipient mice were injected with SEA once to activate T cells (Act) or five times with 4 day intervals to induce tolerance (Tol). Twenty‐four hours after the last injection, CD4^+^TCRVβ3^+^CD45.1^+^ (WT) and CD4^+^TCRVβ3^+^CD45.2^+^ (Egr2/3^−/−^) cells were isolated and re‐stimulated with SEA loaded dendritic cells in vitro. (B) Proliferation was measured 3 days after re‐stimulation. (C) IL2 in supernatants was measured by ELISA 24 h after re‐stimulation. (D) CD62L and CD44 expression on gated CD4^+^TCRVβ3^+^CD45.1^+^ (WT) and CD4^+^TCRVβ3^+^CD45.2^+^ (Egr2/3^−/−^) cells from recipient mice was analyzed 24 h after the last SEA injection. (E and F) IFNγ producing cells among gated CD4^+^TCRVβ3^+^ cells were analyzed after re‐stimulation for 24 h. Data in A, D, E are from five mice in each group and are representative of two experiments. Data in B, C and F are from five mice for each group and are representative of two experiments. Data in B, C, and F are the mean ± s.e.m. and were analyzed with Kruskal–Wallis tests followed by Conover tests with Benjamini–Hochberg correction. N.S. not significant, *p < 0.05, **p < 0.01.

To exclude the possibility of environmental factors affecting tolerance induction in CD2‐Egr2/3^−/−^ mice, irradiated wild‐type mice were reconstituted with a mixture of bone marrow from wild‐type and CD2‐Egr2/3^−/−^ mice (Fig. 5A) as described previously 14. In vivo T cell tolerance was induced in these chimeras by repeated SEA administration. Wild‐type TCRVβ3^+^CD4^+^ cells in the chimeras displayed proliferative and inflammatory tolerance after tolerogenic treatment with SEA (Fig. 5B–F). Consistent with the results from CD2‐Egr2/3^−/−^ mice, Egr2/3 deficient TCRVβ3^+^CD4^+^ cells in the chimeras also displayed similar impairments in proliferation and IL2 production (Fig. 5B and C), but expressed high levels of activation markers and produced excessive levels of inflammatory cytokines such as IFNγ (Fig. 5D–F). These results demonstrated that Egr2 and 3 intrinsically control the inflammatory responses, but not proliferation, of tolerant T cells.

### Egr2 and 3 regulate inflammatory repressors and the function of T‐bet in tolerant T cells

**Figure 6:**
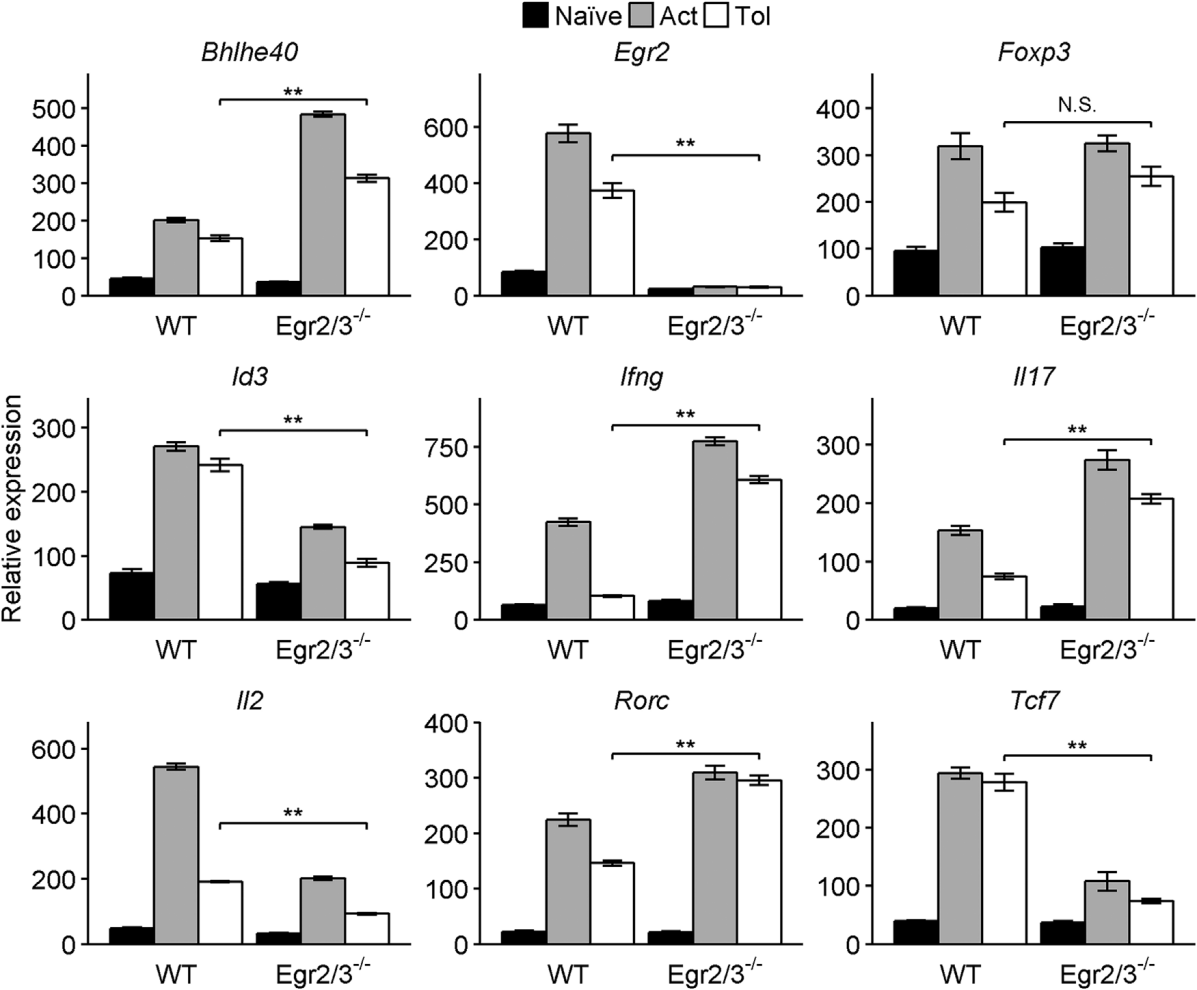
Transcription factors regulated by Egr2 and 3 during immune responses are altered in Egr2/3 deficient tolerant T cells. Naïve CD4 T cells (CD62L^+^CD44^lo^) from wild‐type (WT) and CD2‐Egr2/3^−/−^ (Egr2/3^−/−^) mice were stimulated with anti‐CD3 alone for 24 h, then washed and rested for 24 h before re‐stimulation with anti‐CD3 and anti‐CD28 (Tol). After 24 h expression of transcription factors and cytokines was analyzed and compared to naïve and activated T cells (Act). Data were analyzed using the ddCt method with GAPDH as a reference gene and are representative of three independent experiments. Data are the mean ± s.e.m. and were analyzed with Kruskal–Wallis tests followed by Conover tests with Benjamini–Hochberg correction. N.S. not significant, *p < 0.05, **p < 0.01.

T cell tolerance is defined experimentally by impaired proliferation and production of IL2 in response to antigen stimulation 2. Although Egr2 and 3 are induced in tolerant T cells by antigen stimulation 7, 8, their function in effector T cells is to promote antigen mediated proliferation, but suppress inflammatory differentiation 11, 14. The mechanisms of Egr2 and 3 function in the control of effector T cells in adaptive immunity are to regulate the expression of inflammatory repressors and to directly inhibit the function of T‐bet 13, 14. To investigate the mechanisms of Egr2 and 3 function in the control of inflammatory responses of tolerant T cells, anergy was induced in naïve CD4 T cells from wild‐type and CD2‐Egr2/3^−/−^ mice in vitro and the expression of repressors of effector differentiation (Id3 and Tcf1, encoded by the *Tcf7* gene) regulated by Egr2 and 3 14 was analyzed. Indeed, consistent with the high levels of inflammatory cytokines, the expression of repressors of T cell differentiation was significantly reduced in Egr2/3^−/−^ tolerant T cells (Fig. 6). In contrast, transcription factors involved in differentiation (Bhlhe40 and RORγt, encoded by the *Rorc* gene) were increased (Fig. 6). The altered expression of transcription factors that regulate inflammation was associated with impaired IL2 and increased IFNγ and IL17 expression in Egr2/3^−/−^ tolerant T cells (Fig. 6). Importantly, FoxP3 expression in wild‐type and Egr2/3^−/−^ tolerant T cells was similar (Fig. 6), indicating that these differences are not due to defective FoxP3 expression consistent with our previous findings 11. These results demonstrate that Egr2 and 3 function via similar mechanisms to inhibit inflammatory responses of effector 14 and tolerant T cells.

**Figure 7:**
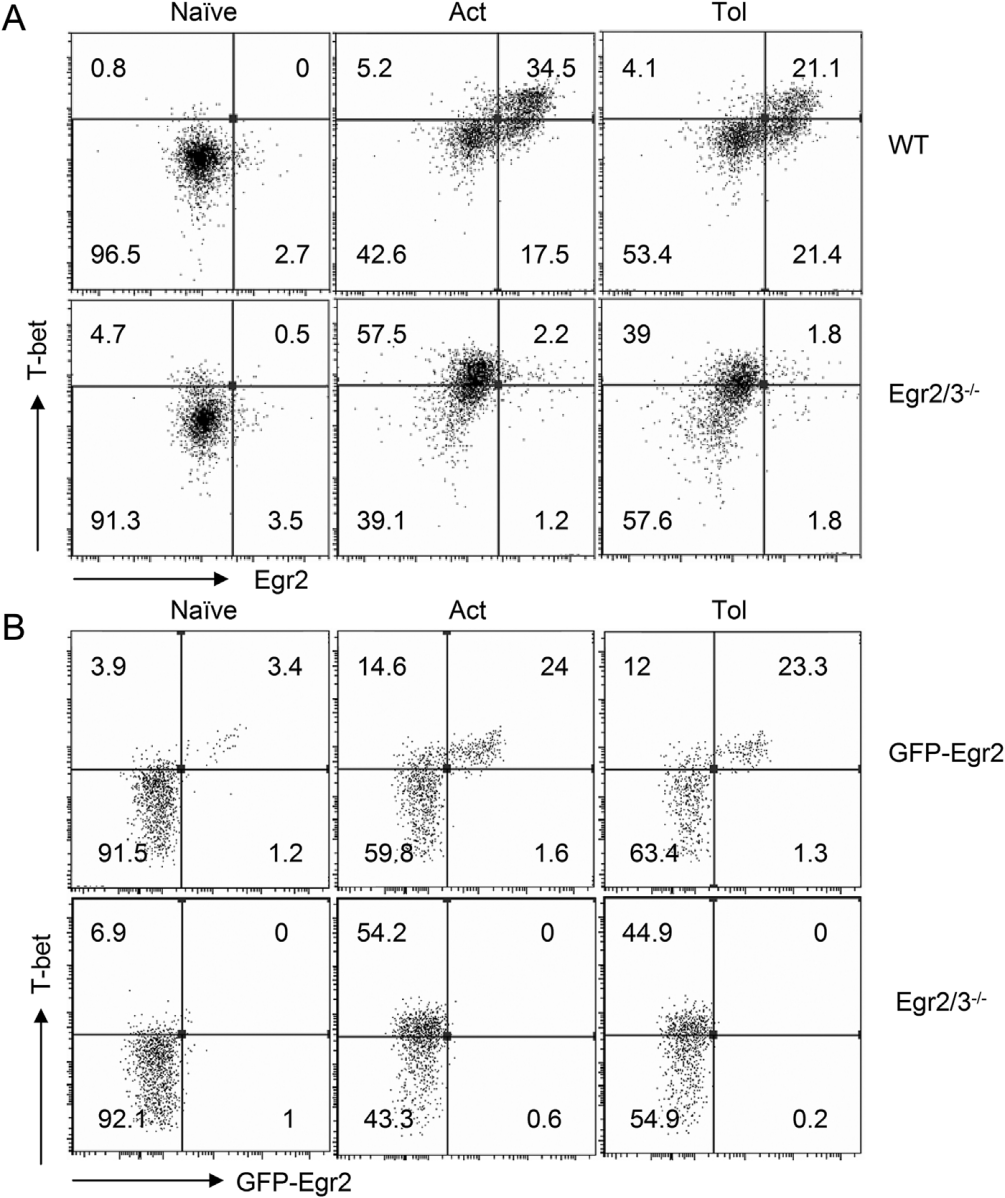
T‐bet is co‐expressed with Egr2 in tolerant T cells. (A) Naïve CD4 T cells (CD62L^+^CD44^lo^) from wild‐type (WT) and CD2‐Egr2/3^−/−^ (Egr2/3^−/−^) mice were stimulated with anti‐CD3 alone for 24 h, then washed and rested for 24 h before re‐stimulation with anti‐CD3 and anti‐CD28 (Tol). After 24 h expression of T‐bet and Egr2 was analyzed and compared to naïve and activated T cells (Act). (B) GFP‐Egr2 knockin and CD2‐Egr2/3^−/−^ mice were injected with SEA once to activate T cells (Act) or five times with 4 day intervals to induce tolerance (Tol). Twenty‐four hours after the last injection, CD3^+^CD4^+^TCRVβ3^+^ cells were analyzed for T‐bet and Egr2 expression. Data in A are from pooled cells of three mice and are representative of three experiments. Data in B are from four mice in each group and are representative of two experiments.

We previously found that Egr2 can directly inhibit T‐bet function 13. However, whether T‐bet is induced in tolerant T cells in response to TCR stimulation is unknown. We discovered that T‐bet was induced in both activated and anergic wild‐type T cells and T‐bet was co‐expressed with Egr2 (Fig. 7A). Moreover, T‐bet was highly induced in both activated and anergic Egr2/3 deficient T cells (Fig. 7A). Similarly, co‐expression of Egr2 and T‐bet was detected in TCRVβ3^+^CD4^+^ cells from GFP‐Egr2 knockin mice following activating or tolerizing treatment with SEA in vivo (Fig. 7B). Thus, consistent with the inhibitory effect of Egr2 and 3 on T‐bet function in adaptive immune responses 13, Egr2 and 3 may control T‐bet function in tolerant T cells.

## Discussion

Effector phenotype T cells with high levels of activation markers such as CD44 accumulate during homeostatic responses 17, 18. However, these cells maintain their tolerance to self‐antigens which is regulated by both extrinsic and intrinsic mechanisms 19, 20. Two experimental systems have been established for investigation of T cell tolerance, T cell anergy induced by suboptimal TCR stimulation in vitro 2 and T cell tolerance induced by repetitive stimulation with high affinity antigens in vivo 7, 21. The hallmarks of tolerance in both systems are impaired proliferation and IL2 production in response to antigen stimulation 2, 7, 21. Egr2 and 3 were initially discovered in tolerant T cells following antigen stimulation in vitro and in vivo 7, 8 and were suggested to negatively regulate the activation of T cells 9. However, recent findings demonstrate that Egr2 and/or 3 are not inhibitors, but promoters, of TCR mediated proliferation and IL2 production 11, 14. Here, we demonstrated a novel function of Egr2 and 3 in tolerant T cells: suppressing inflammatory responses.

Although impaired proliferation and production of IL2 in response to antigen stimulation are the hallmarks of T cell tolerance, in many, if not all, autoimmune diseases, T cells are hyper‐activated and display unwanted effector function, but their proliferation and production of IL2 are impaired 4. This phenotype is associated with defects in TCR signaling 4, suggesting that impaired TCR signaling cannot drive cell cycle progression, but can still induce activation and effector function of tolerant T cells. The increased inflammatory activation, yet impaired proliferation, of Egr2/3^−/−^ tolerant T cells demonstrates that the development of effector function may not be controlled by same mechanisms that control cell cycle progression and production of IL2, and that the effector function of tolerant T cells is controlled at least partly by Egr2 and 3.

In contrast to the impaired induction of AP1 and NFkB in tolerant T cells 2, 7, 21, transcription factors important for regulating the differentiation of effector T cells were induced by TCR stimulation in both activated and tolerant T cells. Egr2 and 3 are important regulators that control expression of inflammatory transcription factors, such as RORγt and Bhlhe40, while inducing expression of repressors of inflammatory differentiation, such as Tcf1 and Id3, in effector T cells 14. Deficiency of Egr2 and 3 leads to altered expression of these transcription factors and severe inflammatory pathology during viral infection 14. We have now demonstrated that the Egr2/3 regulatory pathway is also important to control inflammatory responses of tolerant T cells by a similar mechanism. Thus, deficiency in Egr2 and 3 does not prevent the induction of proliferative tolerance, but causes tolerant T cells to become activated and produce inflammatory cytokines, a phenotype resembling effector T cells from autoimmune diseases such as lupus 4.

Th1 inflammatory responses are a major mechanism in most autoimmune diseases and T‐bet plays an important role in Th1 type autoimmune responses 22, 23. Despite the evidence of increased T‐bet function in autoimmunity, the mechanism leading to inappropriate T‐bet mediated Th1 responses in these autoimmune conditions is unknown. We have recently discovered that Egr2 can directly inhibit T‐bet function in effector T cells 13 and have now found that T‐bet is expressed in tolerant T cells. The expression of T‐bet in tolerant T cells indicates that although the TCR mediated biochemical signals are insufficient for inducing T cells to enter the cell cycle, the signal is enough to induce expression of T‐bet. T‐bet in tolerant T cells is co‐expressed with Egr2 and defects in Egr2 and 3 leads to high levels of IFNγ production in tolerant T cells. These results demonstrate the importance of inhibition of T‐bet by Egr2 and 3 13 in tolerant T cells and suggest that Egr2 and 3 control Th1‐like inflammatory responses in homeostatic conditions, which may be an important mechanism for the control of autoimmunity.

Recently, conflicting findings on the role of Egr2 and 3 in regulation of T cell proliferation have been reported 8, 9, 11. The findings that Egr2 is induced in tolerant T cells and forced Egr2 expression leads to inhibition of proliferation in vitro after TCR stimulation suggest a function of Egr2 in the induction of proliferative tolerance 9. However, it has been reported that forced expression of Egr2 in cell lines of many tissue types results in growth inhibition 24, which indicates an artificial effect of Egr2 overexpression. In contrast, peripheral T cells from Egr2 or Egr3 single deficient mice display weak proliferative responses or relatively normal responses to TCR stimulation in vitro or viral infection 10, 11, 25, 26. The severely impaired proliferation of T cells from CD2‐specific Egr2/3^−/−^ mice demonstrates that Egr2/3 are essential for antigen mediated T cell proliferation 11. We have now demonstrated that Egr2 and 3 are not required for the induction of proliferative tolerance, but play a key role in the control of inflammatory responses of T cells under tolerogenic conditions.

Our findings of separate mechanisms controlling proliferation and effector function of tolerant T cells open potential new strategies for modulating T cell function in tumor immunology and autoimmunity to adjust proliferation and effector function individually.

## Authors' Contributions

PW and SL conceived and coordinated the study. PW wrote the paper. BO, TM, AS, RS, SL, and PW designed, performed and analyzed experiments.

## Conflict of Interest

The authors declare no commercial or financial conflict of interest.
